# Draft Genome Sequence of *Bacillus thuringiensis* Serovar Tolworthi Strain Na205-3, an Isolate Toxic for *Helicoverpa armigera*

**DOI:** 10.1128/genomeA.00187-14

**Published:** 2014-03-13

**Authors:** Leopoldo Palma, Delia Muñoz, Jesús Murillo, Primitivo Caballero

**Affiliations:** a Instituto de Agrobiotecnología, CSIC-UPNA-Gobierno de Navarra, Campus Arrosadía, Mutilva Baja, Navarra, Spain; b Departamento de Producción Agraria, Universidad Pública de Navarra, Pamplona, Navarra, Spain

## Abstract

We report here the complete annotated 6,510,053-bp draft genome sequence of *Bacillus thuringiensis* serovar tolworthi strain Na205-3, which is toxic for *Helicoverpa armigera*. This strain potentially contains nine insecticidal toxin genes homologous to *cry1Aa12*, *cry1Ab1*, *cry1Ab8*, *cry1Ba1*, *cry1Af1*, *cry1Ia10*, *vip1Bb1*, *vip2Ba2*, and *vip3Aa6*.

## GENOME ANNOUNCEMENT

*Bacillus thuringiensis* (*Bacillales*: *Bacillaceae*), one of the best-characterized entomopathogenic bacteria, carries plasmids bearing a variety of genes coding for proteins with valuable insecticidal characteristics (1). These proteins can be classified into distinct protein groups according to their amino acid identities and protein structures: Cry and Cyt proteins (δ-endotoxins) (2); vegetative insecticidal proteins Vip1/Vip2 (binary toxin) (3), Vip3 (4), and Vip4 (http://www.lifesci.sussex.ac.uk/Home/Neil_Crickmore/Bt/); Bin-like and Mtx-like proteins (1, 5); the secreted insecticidal proteins Sip (6); accessory proteins P19 and P20 (7); and enhancin-like proteins (8). In this work, we report the draft genome sequence of *Bacillus thuringiensis* serovar tolworthi strain Na205-3, which is toxic against *Helicoverpa armigera* (Lepidoptera: Noctuidae) (9). Purified total DNA from strain Na205-3 was sequenced at the Beijing Genomics Institute (BGI) (Shenzhen, China) using high-throughput Illumina sequencing technology. The reads were assembled using SOAP*denovo* (version 1.05) and produced 169 contigs totaling 6,510,053 bp, with a maximum scaffold size of 427,627 bp, an *N*_50_ length of 161,129 bp, and 34.7% G+C content. Genome annotation was added by the NCBI Prokaryotic Genome Annotation Pipeline (released in 2013), although it was also analyzed with BLAST (10) using a custom insecticidal toxin database, the RAST server (11), and the BtToxin_scanner (12). The custom insecticidal toxin database was constructed with amino acid sequences of Cry and Cyt proteins, Bin-like and Mtx-like proteins, Vip1/Vip2, Vip3, and Sip1A proteins (13), accessory proteins P19 and P20, and the enhancin-like Bel protein. The Na205-3 draft genome sequence carries nine insecticidal toxin genes homologous to *cry1Aa12*, *cry1Ab1*, *cry1Ab8*, *cry1Ba1*, *cry1Af1*, *cry1Ia10*, *vip1Bb*1, *vip2Ba2*, and *vip3Aa6*, a putative *etx_mtx* toxin gene, and 1 *bel* (enhancin-like) gene. This ample repertoire of insecticidal genes implies that strain Na205-3 might have a wider host range than was previously thought.

### Nucleotide sequence accession numbers.

This whole-genome shotgun project has been deposited at DDBJ/EMBL/GenBank under the accession no. AYXQ00000000. The version described in this paper is version AYXQ01000000.
